# Effect of vaginal self-sampling on cervical cancer screening rates: a community-based study in Newfoundland

**DOI:** 10.1186/s12905-015-0206-1

**Published:** 2015-06-10

**Authors:** Pauline Duke, Marshall Godwin, Samuel Ratnam, Lesa Dawson, Daniel Fontaine, Adrian Lear, Martha Traverso-Yepez, Wendy Graham, Mohamad Ravalia, Gerry Mugford, Andrea Pike, Jacqueline Fortier, Mandy Peach

**Affiliations:** Primary Healthcare Research Unit, Memorial University, St John’s, Canada; Discipline of Family Medicine, Memorial University, Newfoundland & Labrador, Primary Healthcare Research Unit, Room 424, Janeway Hostel, 300 Prince Philip Drive, St. John’s, A1B 3 V6 NL Canada; Public Health Laboratories, Eastern Health Authority, St. John’s, Canada; Department of Obstetrics and Gynecology, Memorial University, St. John’s, Canada; Department of Pathology, Eastern Health Authority, St. John’s, Canada; Cancer Clinic, Eastern Health Authority, St. John’s, Canada; Community Health and Humanities, Memorial University, St. John’s, Canada; Department of Psychiatry, Memorial University, St. John’s, Canada

**Keywords:** Cervical cancer screening, Women’s health, Community medicine, Health promotion, Patient education

## Abstract

**Background:**

Cervical cancer is highly preventable and treatable if detected early through regular screening. Women in the Canadian province of Newfoundland & Labrador have relatively low rates of cervical cancer screening, with rates of around 40 % between 2007 and 2009. Persistent infection with oncogenic human papillomavirus (HPV) is a necessary cause for the development of cervical cancer, and HPV testing, including self-sampling, has been suggested as an alternative method of cervical cancer screening that may alleviate some barriers to screening. Our objective was to determine whether offering self-collected HPV testing screening increased cervical cancer screening rates in rural communities.

**Methods:**

During the 2-year study, three community-based cohorts were assigned to receive either i) a cervical cancer education campaign with the option of HPV testing; ii) an educational campaign alone; iii) or no intervention. Self-collection kits were offered to eligible women at family medicine clinics and community centres, and participants were surveyed to determine their acceptance of the HPV self-collection kit. Paired proportions testing for before-after studies was used to determine differences in screening rates from baseline, and Chi Square analysis of three dimensional 2 × 2 × 2 tables compared the change between communities.

**Results:**

Cervical cancer screening increased by 15.2 % (*p* < 0.001) to 67.4 % in the community where self-collection was available, versus a 2.9 % increase (*p* = 0.07) in the community that received educational campaigns and 8.5 % in the community with no intervention (*p* = 0.193). The difference in change in rates was statistically significant between communities A and B (*p* < 0.001) but not between communities A and C (*p* = 0.193). The response rate was low, with only 9.5 % (168/1760) of eligible women opting to self-collect for HPV testing. Of the women who completed self-collection, 15.5 % (26) had not had a Pap smear in the last 3 years, and 88.7 % reported that they were somewhat or very satisfied with self-collection.

**Conclusions:**

Offering self-collected HPV testing increased the cervical cancer screening rate in a rural NL community. Women who completed self-collection had generally positive feelings about the experience. Offering HPV self-collection may increase screening compliance, particularly among women who do not present for routine Pap smears.

## Background

Cervical cancer is preventable and treatable if detected early. Persistent infection with oncogenic human papillomavirus (HPV) is a necessary cause of cancer [1, 2]. While most HPV infections are transient and clear in 12 to 18 months [3, 4], women who have persistent infection with oncogenic HPV are at increased risk of pre-cancerous lesions and cervical cancer. Asymptomatic cervical HPV infection can be detected in 5–40 % of women of reproductive age with the prevalence declining with advancing age [5]. The relative risk for the association between HPV infection and cervical neoplasia is of high magnitude, typically in the 20–70 range [5]. This range is greater than that for the association between smoking and lung cancer and is comparable only to that of the association between chronic hepatitis B infection and liver cancer [5].

In countries where Pap smear screening is widely available, studies have shown that more than half of all invasive cervical cancers are diagnosed in women who are under- or un-screened [6, 7]. These studies have also suggested that up to 30 % of women diagnosed with invasive cervical cancer were considered adequately screened by Pap smear [6, 7]. Self-collected HPV testing has been suggested as an effective cervical cancer screening tool that is acceptable to women and may encourage better adherence to regular screening, particularly among women who are under-screened [8–10]. Studies have demonstrated that detecting early cell changes earlier using HPV does prevent some cancers [11–13] and women report satisfaction with self-sampling and a willingness to screen in this manner [14]. Self-sampled HPV testing alleviates some of the discomfort and embarrassment that some women feel with a Pap smear, which may be a barrier to screening [15, 16]. The use of HPV testing as a primary screening tool is still the subject of debate in the literature. The Canadian Task Force for Preventive Health Care felt there was insufficient evidence to endorse HPV testing as a method of primary cervical cancer screening in its 2013 recommendations [17], but a self-sampled HPV test has been approved as a primary screening tool in the US [18] and many studies continue to evaluate the potential of this technology in low-resource settings [19, 20].

It has long been known that individuals living in rural communities have poorer prognosis for a variety of health conditions [21], including an adjusted cancer survival rate that is 5–7 % lower than their urban-dwelling peers [22]. Referral delays, lack of providers, providers’ attitudes toward screening [23], and limited access to screening [24] are cited as potential reasons for this disparity. Self-sampled HPV testing has been studied as a way to screen women for cervical cancer in rural parts of developing countries [25, 26], but its efficacy and effectiveness in rural and remote regions of developed nations is less understood.

Taking this into consideration, we devised a community-based study in rural areas of Newfoundland and Labrador (NL), Canada, to determine whether offering self-collected HPV testing might be an effective tool to increase cervical cancer screening participation. Despite comprehensive education programs and promotional campaigns, only 43 % of women in NL participated in Pap smear screening from 2007–2009 [27]. Our objective was to determine if the introduction of a self-collection strategy for HPV screening would increase primary cervical cancer screening in a community-based setting in rural NL.

## Method

### Study design

**Fig. 1:**
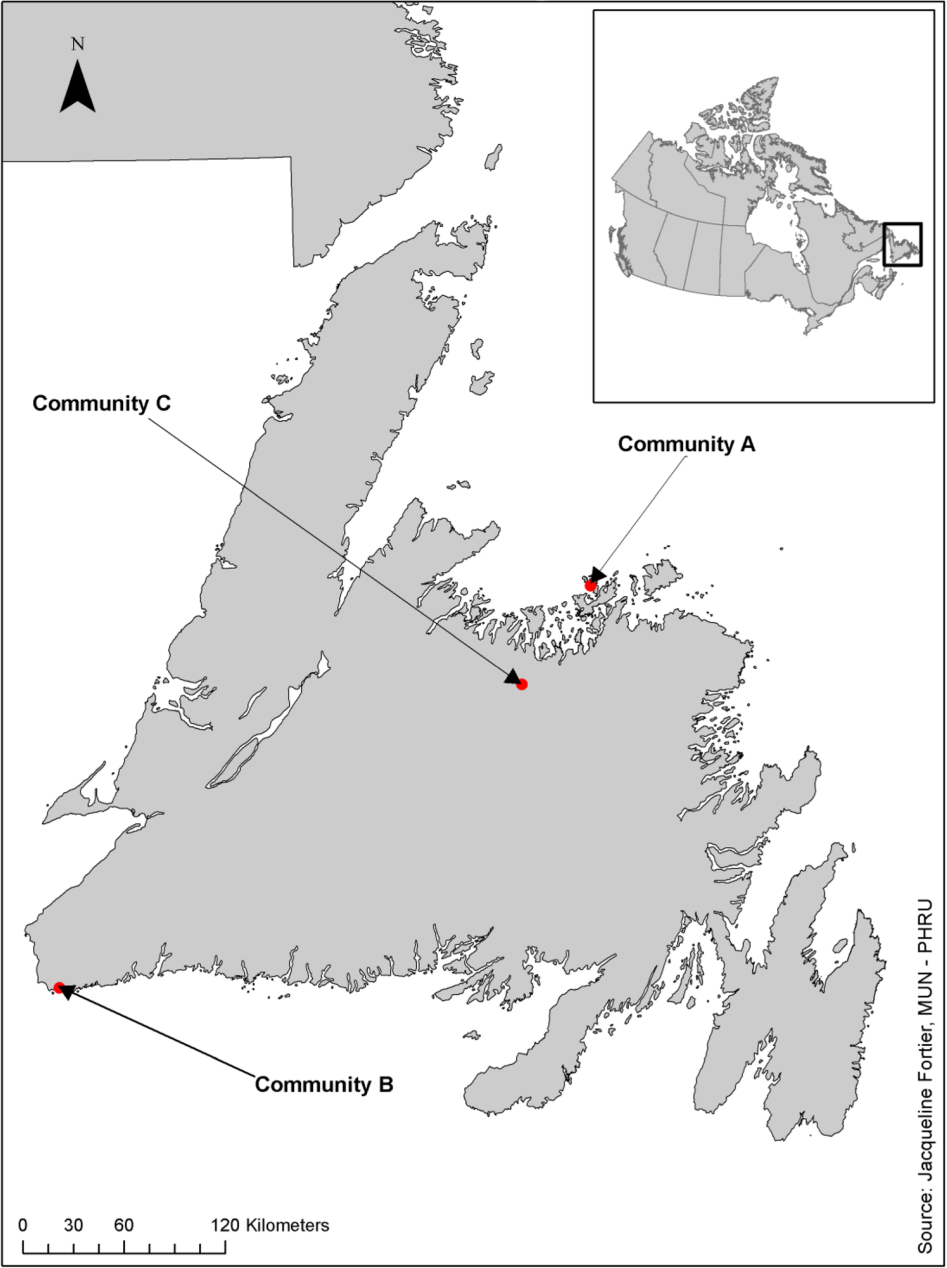
Approximate location of the three study communities on the island of Newfoundland

This study used a community-based cohort design targeting women aged 30–69 years, excluding pregnant women. Three communities in NL with similar demographics (Table 1) that were far enough apart to avoid contamination were identified (minimum 140 km or 86 mi by road, see Fig. 1). The two intervention communities (Communities A and B) were rural communities of similar size and demographic composition with the presence of academic faculty members to oversee the research. The control community was selected because it was demographically similar to the two study communities. The eligible study populations before and after the intervention, used to calculate participation rates, were determined using census data from the 2006 to 2011 Canadian census [28, 29].

**Table 1:** Population and demographics for the study communities at beginning of study based on 2006 data

Community	Community catchment area population	N (%) of women aged 30–69 in community catchment area	Personal income per capita	Employment rate	Pap smear rates
A	6280	1928 (30.7 %)	$16,900	75.2 %	41 %
B	6475	2833 (43.8 %)	$18,400	70.4 %	38 %
C	5355	1524 (28.6 %)	$16,600	63.3 %	45 %

These are three rural communities around the coast of the island of Newfoundland, Canada. There locations around the island are shown in Fig. 1. Fishing, logging, water based transportation, and services industries are the main sources of employment. The people are almost exclusively Caucasian

Potential participants living in Community A were given the option of being screened for HPV infection through a vaginal self-collection method and were also encouraged to undergo a Pap smear screening test done by their primary health care provider; Communities B and C had the continued availability of Pap smears for cervical cancer screening (Table 2). Educational and promotional activities in Communities A and B raised awareness about the prevalence and preventability of cervical cancer, the importance of regular screening. In Community A this promotional campaign included information about the availability of self-collected HPV testing offered as part of a research project while in Community B the focus was on the importance of Pap smears. Community C received no intervention beyond the normal public education initiatives conducted by the provincial cervical screening program. Research nurses carried out all educational activities in Communities A and B.

**Table 2:** Screening and educational interventions in the three communities included in the study

	Community A	Community B	Community C
Screening	HPV self-collection in addition to regular Pap test screening	Regular Pap test screening	Regular Pap test screening
Education	Intense educational and promotional campaign about HPV, self-collection and cervical cancer screening in addition to regular provincial education campains	Intense educational and promotional campaign about cervical cancer screening in addition to regular provincial education campaigns	Regular provincial education campaigns

The Newfoundland and Labrador Human Research Ethics Board reviewed and approved the study. All participants provided informed consent prior to enrollment.

### Self-collection kit

**Fig. 2:**
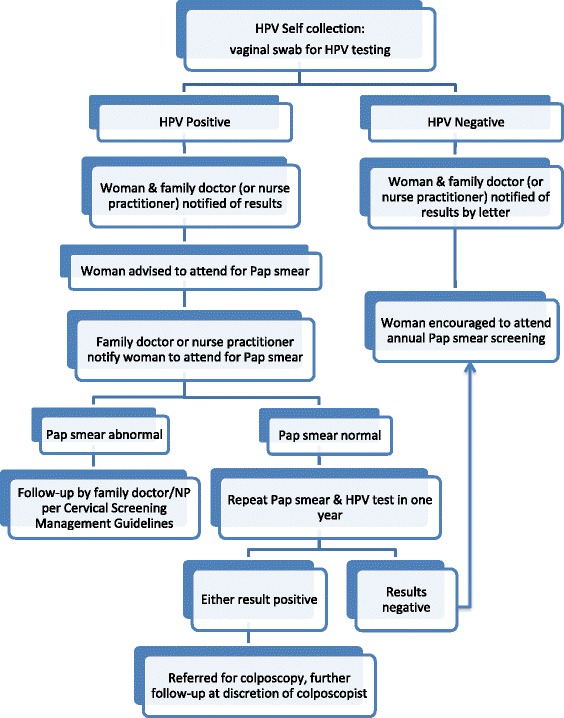
Flow sheet algorithm for follow-up of HPV test results

The HPV self-collection kit was assembled by the Newfoundland & Labrador Public Health Laboratory and included a Dacron swab, collection tube, instructions with explanatory pictures, consent forms and a participant questionnaire examining demographic information, knowledge of Pap smear screening, frequency of screening, and satisfaction with the self-collection process. Kits were available at public locations such as the hospital, pharmacies, hair salons and women’s exercise centers. The research nurse was also available to drop off kits at a woman’s home or work. Once the kit was completed women could drop it off at the local hospital or call the research nurse and she would pick up the kit at a place and time of their convenience. The kits were sent to the Public Health Laboratory in St. John’s, NL for HPV DNA testing within 2 weeks of sample collection. All participating women signed written consent for involvement in the study. Patients and their primary care providers were contacted to provide the results of the HPV testing and suggestions of the next steps (Fig. 2). Women who tested negative for HPV were given a pamphlet explaining the importance of continuing regular screening for cervical cancer while women who were positive for HPV were given an informational pamphlet about HPV and a recommendation to follow-up with their primary care provider for Pap smear screening.

### Educational and promotional campaigns

The province of NL has a Cervical Screening Initiative promotes cervical cancer screening through regular educational and promotional activities. The Initiative distributes informational pamphlets and posters, provides educational sessions in the community, and promotes cervical cancer awareness and screening through radio and newspaper advertisements. The Initiative also has an opt-in program where primary caregivers are notified when their patients are due to be screened by Pap smear. Regional personnel oversee the Initiative’s promotional activities, and individual primary care providers may have their own strategies to promote screening among their patients.

The research nurse in Communities A and B gave presentations on cervical cancer screening to various community groups as part of an educational campaign. Women in Community A were also given the option to take a self-collection kit at the conclusion of the presentation. In Community A the research nurse held drop-in clinics where women could pick up or drop off kits, get help filling out paperwork or ask any questions they may have. In Community B, participating women completed a questionnaire about Pap smear screening at the time of a scheduled Pap smear or after attending an educational session. In Communities A and B the campaign to promote screening through HPV self-collection or Pap smear, respectively, was promoted through radio ads, newspaper ads and community newsletters, and other local media and community groups (Table 3). The degree of penetration of the promotional and educational campaigns was assessed via telephone survey of 15 % of non-commercial listed phone numbers approximately a year after the study began.

**Table 3:** Promotional activities carried out in Community A and Community B

Category	Community A	Community B
Newspaper ads^a^	15	13
Online newspaper ads^a^	5	5
Newspaper articles^a^	2	1
Public Service Ads^a^	48	55
Radio Ads^a^	7	7
Radio Interview	1	−
Community Newsletters^a^	4	1
Church Bulletins	20	−
Facebook Page	1	1
Facebook Advertising	1	1
Digital Community Sign^a^	−	4
Nurse Presentations	37 presentations	34 presentations
	17 promotions	
	44 drop-in clinics	

^a^For these activities, the number represents the number of times the promotion was carried out over a 1 week period. For example, in Community A, ads were put in the local newspaper 15 times for a total of 15 weeks in circulation

### Statistical analysis

Demographics were described for study participants in each region, with age, marital status, and level of education grouped categorically. The cervical cancer screening history of participants in Communities A and B was assessed to categorize their screening status as regularly screened (had Pap smear in past 3 years), under-screened (last Pap smear between 3 and 5 years ago) or unscreened (never had a Pap smear).

The cervical cancer screening rates were calculated for each community for the 2 years prior to the study (2008 and 2009) and the two years the study was in place (2010 and 2011). Pap smear data was provided by the provincial Cervical Screening Initiatives Program. For Community A the cervical cancer screening rate included those screened by Pap smear or by self-collection for HPV screening; individuals who were screened by self-collection and Pap smear during the study period were identified and counted only once. For Communities B and C the screening rates were calculated using provincial Pap smear screening data. Paired proportions testing for Before-After studies was used to determine the statistical significance of the change in screening rates for each community. We then compared the change in screening rates between Community A and each of Community B and Community C using Chi Square analysis of 3-dimension 2 × 2 × 2 tables to determine if the intervention resulted in significantly different changes in screening rates in the intervention community compared to the control communities.

## Results

### Study demographics and community participation

There were an estimated 1928 women aged 30–69 years of age in Community A, 2833 in Community B, and 1524 in Community C at the beginning of the study. By the end of the study the populations of eligible women in Communities A, B and C were 1760, 2761, and 1536 respectively.

Eight hundred and thirty seven HPV self-collection kits were taken from the distribution sites in Community A during 2010 and 2011, and 168 (20.1 %) of these were returned for processing. The overall response rate of eligible women to self-collection in Community A was 9.5 % (168/1760). In Community B, 374 (13.5 %) women presenting for Pap smears agreed to be part of the study and provided information on themselves and their screening history.

The participants in Communities A and B were similar demographically, although Community A had significantly more women with less than a high school education and fewer women with a college degree (Table 4). A majority of participants in both communities were married and had at least some post-secondary education.

**Table 4:** Demographics of Participants in Community A and Community B

Variable	Category	Community A (*n* = 168) n(%)	Community B (*n* = 374) n(%)
Age Group	30–34	14 (8.3 %)	34 (9.1 %)
	35–39	9 (5.4 %)*	54 (14.4 %)*
	40–44	22 (13.1 %)	46 (12.3 %)
	45–49	29 (17.3 %)	58 (15.5 %)
	50–54	31 (18.5 %)	59 (15.8 %)
	55–59	32 (19 %)	55 (14.7 %)
	60–64	21 (12.5 %)	34 (9.1 %)
	65–69	6 (3.6 %)	21 (5.6 %)
	unknown	4 (2.4 %)	13 (3.5 %)
Marital Status	Married or Common Law	148 (88.1 %)	310 (82.9 %)
	Divorced	3 (1.78 %)	10 (2.7 %)
	Separated	2 (1.19 %)	13 (3.5 %)
	Widowed	4 (2.38 %)	14 (3.7 %)
	Single or Never Married	9 (5.36 %)	19 (5.1 %)
	Unknown	2 (1.19 %)	8 (2.1 %)
Education	< High school	49 (29.17 %)*	59 (15.8 %)*
	High school diploma	44 (26.19 %)	100 (26.7 %)
	Some College	6 (3.57 %)*	32 (8.6 %)*
	College Diploma or Certificate	45 (26.79 %)	114 (30.5 %)
	Some University	6 (3.57 %)	16 (4.3 %)
	University Degree, Diploma, or Certificate	17 (10.11 %)	45 (12.0 %)
	Unknown	1 (0.6 %)	8 (2.1 %)
Pap smear screening history	Regularly screened	142 (84.5 %)	322 (85.9 %)
	Under screened	25 (14.9 %)	39 (10.4 %)
	Unscreened	1 (0.6 %)	13 (3.5 %)*
	Unknown	2 (1.2 %)	1 (0.3 %)

* denotes significant differences in proportions at a significance level of 0.05

The telephone survey assessing the penetration of the educational and promotional campaigns indicated that well over half of the eligible women in Community A (167/276, 74.6 %) and just under a third of eligible women in Community B (117/381, 30.7 %) were aware of the cervical cancer research study in their area.

### Cervical cancer screening history, participation rate, HPV positivity and participant satisfaction

Twenty-six of the women (15.5 %) who completed self-collection in Community A and 52 women (13.9 %) who completed a questionnaire in Community B were under- or un-screened by our definitions. In Community A, 40 women (23.8 %) used self-collection as their only form of cervical cancer screening during the study period. Seven (4.17 %) study participants in Community A tested positive for HPV. Of the women who tested positive for HPV and followed up with the recommended Pap smear, two were found to have a low-grade squamous intraepithelial lesion, one of whom was under-screened.

Overall, participant satisfaction with the kits was high, with 114 (67.9 %) participants stating they were very satisfied with the self-collected HPV test, 35 (20.8 %) were somewhat satisfied and 9 (5.4 %) were uncertain. Only 1 (0.6 %) woman was somewhat dissatisfied and no women were very dissatisfied with the kit. One hundred and fifty three women (91.1 %) said they would use the kit again to regularly screen for HPV.

### Change in cervical cancer screening rates

There was a statistically significant increase in screening rates in Community A (15.2 % increase; *p* < 0.001) and Community C (8.5 % increase; *p* <0.01) during the 2010/2011 period compared to the 2008/2009 period (Table 5). The 2.9 % change in screening rates for Community B was not statistically significant. The change in screening rate for Community A was significantly greater than the change in screening rate in Community B (*p* <0.001). The change in screening rate in Community A was not statistically different from the rate in Community C (*p* = 0.193).

**Table 5:** Rate of cervical cancer screening in the three communities prior to and during the study

Community	Cervical cancer screening rate 2008/2009 n/N^a^ (%)	Cervical cancer screening rate 2010/2011 n/N^a^ (%)	Change in rate from 2008/2009 to 2010/2011	P Value for before-after change in each community	P Value for difference in change in rates
A	1020/1928 (52.9 %)	^b^1187/1760 (67.4 %)	+15.2 %	<0.001	Ref.
B	1484/2833 (52.4 %)	1529/2761 (55.3 %)	+2.9 %	0.07	<0.001
C	1098/1524 (72.0 %)	1236/1536 (80.5 %)	+8.5 %	<0.01	0.193

^a^Denominator (eligible populations of women age 30–69 years) for the 2008/2009 period is based on 2006 census data and for the 2010/2011 period it is based on 2011 census data

^b^For years 2010 and 2011 for Community A the screening rate was determined by adding the number of women who had a Pap smear to the women who did self-collection but did not have a Pap smear to avoid counting in duplicate those women who self-collected and had a Pap smear

## Discussion

### Screening rates

In Community A the cervical cancer screening rate (Pap smear or HPV testing as screen) increased by 15.2 % during the 2 years of the study. This is statistically and, we believe, clinically significant. In Community B the rate increased by only 2.9 % during the study period. This suggests that the availability of self-collection in Community A did improve cervical cancer screening rates beyond the effect of simply having an intense educational and media campaign.

Despite a comparable screening rate of 45 % in 2006, Community C had a screening rate of 72 % in the two years prior to the study, and the screening rate increased by 8.5 % (*p* < 0.001) during the study period. Our study was conducted during a period of time when awareness of the low cervical cancer screening rate was increasing. The overall proportion of women considered adequately  screened in the province rose from 68.2 % during 2006–2008 to 74.4 % during 2009–2011, changing NL from a province with one of the lowest participation rates in the country to among the highest in just 3 years [30, 31]. The provincial Cervical Screening Initiatives educational and promotional campaigns continued in all three communities. Through personal communications with a senior physician in the area, we also learned of a nurse practitioner and a young family physician in Community C who both began practicing in the community immediately prior to and during the study period, both of whom were proactive with cervical cancer screening. These confounding factors may be responsible for the unexpected increase in screening rates in our control community.

### Response rate

Our uptake rate was relatively low compared to other studies of HPV self-collection programs. Of the 837 kits that were picked up, only 168 (20.1 %) were returned, and only 9.5 % of the eligible population of women participated in HPV self-collection. Researchers in Mexico completed a trial of women from low socioeconomic status and obtained a response rate of 74.6 % [9]. Another study in rural Mississippi offered under-screened women the opportunity to self-collect for HPV in their homes or to have a pap smear and 64.7 % chose to self-collect for HPV [32]. In both these studies, however, nurses went directly to participants’ homes and helped them with their sample and paperwork. This type of specialized care would no doubt increase participation rates; however in the general population such intervention is not feasible for each and every woman.

Our objective was to evaluate whether the introduction of self-collected HPV kits alongside traditional Pap smears would increase overall screening in the community. This makes our study more comparable to other screening programs in the country where people are responsible for initiating or completing their own testing. For example, colorectal cancer screening initiatives have participation rates of 23 % in Manitoba (2009/10) [33] and 27 % in Ontario (2009/10) [34]. In Newfoundland and Labrador the participation in breast cancer screening was 25.5 % in 2003–04 after 8 years of the program being in place [35]. These numbers are more comparable with our results.

One also wonders why 669 of the 837 kits that were picked up were not returned. Self-collected HPV testing is thought to overcome many of the barriers to participation in Pap smear screening, including pain and discomfort with pelvic exams, difficulty scheduling appointments, transportation issues, and lack of access to primary care providers, and many studies in the literature report much higher response rates than we observed. In the questionnaires, many of our participants stated that the reason they were under-screened was that they simply, “had not gotten around to [having a Pap smear]” (31.3 %). Perhaps the self-collection kit alleviates the barriers of time and scheduling for some women, but clearly there are still some impediments to wider use of the kits. Another potential barrier was the amount of paperwork involved in the study, which may have deterred some women with lower educational levels to take but not return kits, when they realized the amount of paperwork involved. The kit contained instructions (3 pages), consent forms (6 pages) and questionnaires (3 pages), for a total of 12 pages of documents, which may have been overwhelming or too time-consuming for participants.

The literature suggests that the method by which women are provided with the HPV self-collection kits may affect participation. An Italian study comparing different self-sampling distribution methods found that when kits were mailed directly to women, rather than providing women the option to request a kit, the response rate more than doubled from 8.7 % to 19.6 % [36]. It seems that direct mailing, rather than ‘on demand’ screening may increase participation. As the bulk of the cost of HPV testing is incurred at the laboratory phase, distributing the relatively inexpensive kits widely may, indeed, be cost efficient. The kits used for our study cost $3 CAD each, with an additional cost of $35 CAD plus technician time for the HPV DNA assay at the time of our study, although costs will vary by jurisdiction and are likely to decrease over time. Distributing self-collected HPV kits directly to women identified as under-screened through cervical screening registries may be an efficient method to utilize this technology, and the results from the study mentioned previously indicate a willingness of these women to participate in this way.

Of the women who completed self-collection, 88.8 % found the process somewhat or very satisfactory. Furthermore, 15.5 % (26) of the women who performed the self-collection were under-screened or unscreened. These women did not attend regular screening in the past, and the self-collection kit provided a method that they were more willing to use.

A discussion of cervical cancer screening would be incomplete without the acknowledgement of the importance of organized cervical screening programs. A systematic review of methods to improve cervical cancer screening has found standard recall letters to be effective at increasing compliance with cervical cancer screening via Pap smear [33]. Self-collection has been shown in our study and others to be an effective method of increasing screening rates, but we suggest that it would be most effective if used as part of an organized screening program.

### Implications for rural communities

Self-collection seems to alleviate some barriers to screening in rural communities, but our study indicates that the rate of uptake may hinder its utility. Self-sampling is fast, women are overwhelmingly able to collect adequate samples, and it can be implemented in communities that have no or very few regular primary care providers. However, our study makes clear the fact that simply providing kits may not sufficiently encourage women to self-collect, and further study of more targeted or direct distribution methods is warranted prior to wider use of this self-collected HPV testing.

## Conclusion

Availability of a self-collection option increased the cervical screening rate by 15.2 %. Twenty-six (15.5 %) women who used self-collection in Community A had been unscreened or under-screened prior to the study. Twenty percent of the self-collection kits that were taken from distribution sites were returned with samples. Participants found the self-collection test acceptable. We feel self-collection should be considered as an option for cervical cancer screening as part of an organized cervical screening program.
